# Nutritional Composition, Phytochemical Profiles, and Pharmacological Effects of Ethiopian Eggplant (*Solanum aethiopicum* L.)

**DOI:** 10.3390/nu16234228

**Published:** 2024-12-06

**Authors:** Seung Min Choi, Chang-Ik Choi

**Affiliations:** Integrated Research Institute for Drug Development, College of Pharmacy, Dongguk University-Seoul, Goyang 10326, Republic of Korea; tlsehdhs@dgu.ac.kr

**Keywords:** *Solanum aethiopicum* L., Ethiopian eggplant, African eggplant, scarlet eggplant, pharmacological effects

## Abstract

Natural product therapy has been used to treat illness for thousands of years, and modern-day medicines, such as various anticancer, antihypertensive, and antimigraine drugs, have been developed from natural products. Natural medicines are advantageous as they tend to have fewer side effects and are considered a relatively safe option. *Solanum aethiopicum* L. (*S. aethiopicum*) is a vegetable crop of the Solanaceae family and is considered one of the five most important crops in sub-Saharan Africa, alongside tomatoes, onions, peppers, and okra. *S. aethiopicum* has many health benefits as it contains the three major macronutrients (carbohydrates, proteins, and fats) as well as fiber and many essential vitamins. Additionally, much research has been conducted on the medicinal value of *S. aethiopicum* over the past few decades. *S. aethiopicum* has been found to have many pharmacological properties including anti-inflammatory, antibacterial, antidiabetic, anti-obesity, and antioxidant effects. Currently, to our knowledge, there are no comprehensive reviews of the numerous studies on *S. aethiopicum*. Therefore, in this review, we summarize the nutritional, phytochemical, and pharmacological analyses of *S. aethiopicum*, identify notable effects, and review the results.

## 1. Introduction

Treating illness using natural products has been practiced for thousands of years. The practice continues today despite numerous medical advancements, and the field of drug development utilizing natural products is still actively progressing. In modern times, various types of medicines, such as anticancer, antihypertensive, and antimigraine drugs, have been developed based on natural products [1]. Natural medicines are advantageous as they tend to have fewer side effects and are considered a relatively safe option [2]. According to the World Health Organization (WHO), 80% of the world’s population relies on natural products for medicinal purposes, so traditional medicine systems based on natural products are economically crucial [3].

*Solanum aethiopicum* L. (*S. aethiopicum*), commonly known as Ethiopian eggplant, African eggplant, scarlet eggplant, bitter tomato, or mock tomato, is a vegetable crop of the Solanaceae family, similar in appearance to eggplant and tomato and can be of various types, shapes, and colors [4]. *S. aethiopicum* is native to sub-Saharan Africa and is one of the five most important crops in tropical Africa, alongside tomatoes, onions, peppers, and okra. *S. aethiopicum* produces roughly 25 tons of harvest per hectare, making it a good and dependable crop. *S. aethiopicum* is associated with poor post-harvest handling practices and a short shelf life of about 3–4 days. Therefore, care must be taken to avoid the effects of factors that affect the post-harvest quality of the fruit, such as ripening stage, harvesting method, harvesting time, classification and grading, packaging, storage type, and temperature and humidity during storage [5]. It has also recently been cultivated in parts of the Caribbean, South America, and southern Italy [6,7,8,9]. *S. aethiopicum* belongs to the Solanaceae family, which encompasses hundreds of cultivars that vary by region. In the 1980s, extensive systematic studies conducted by Omidiji in Nigeria and Leicester in the UK revealed a high degree of hybridization among the distinct species, ultimately confirming that they represent a single species: *S. aethiopicum*. Furthermore, it was established that *S. aethiopicum* was domesticated from *Solanum anguivi* Lam. Although this *S. aethiopicum* was initially classified as four distinct species—Gilo, Shum, Kumba, and Aculeatum—they are now all recognized as cultivars of *S. aethiopicum* [10,11,12]. The Gilo and Kumba groups are mainly cultivated for their fruit in the same way as the common eggplant and are cooked in stews or eaten raw [5]. Additionally, along with the Shum group, the Kumba group is also cultivated for its leaves, which are consumed in a form similar to spinach. Finally, the Aculeatum group is cultivated for its rhizomes and for ornamental purposes [5,11]. *S. aethiopicum* contains the three major macronutrients (carbohydrates, proteins, and fats) as well as fiber and many essential vitamins [13,14]. *S. aethiopicum* is considered a nutraceutical and has been extensively studied for its medicinal properties over the past several decades [6,9,13], including its anti-inflammatory, antibacterial, antidiabetic, anti-obesity, and antioxidant effects.

Currently, to our knowledge, there are no comprehensive reviews of the numerous pharmacological studies on *S. aethiopicum*. Therefore, in this review, we summarize the pharmacological analyses of *S. aethiopicum*, identify notable effects, and review the results.

## 2. Methods

This review was conducted by extensively combining various keywords such as “pharmacological effect”, “bioactivity”, “anti-inflammatory effects”, “antidiabetic effects”, “anti-obesity effects”, “anxiolytic effects”, “antibacterial effects”, “anticancer effects”, “antioxidant effects”, “nutritional composition”, “chemical composition”, “bioactive compound”, etc., with “*Solanum aethiopicum*”, “Ethiopian eggplant”, “African eggplant”, and “Scarlet eggplant” as a keyword. Using “*Solanum aethiopicum*” as a keyword, 7090 literature results were found, and 76 research and review publications related to pharmacological effects and *S. aethiopicum* published between 1986 and 2024 were included in this review. In this process, results with the same botanical name or crop name, such as “African eggplant”, but different scientific names from “*Solanum aethiopicum*” were excluded, and other documents with low relevance and potential for confusion were excluded. The articles were collected through various sources, such as MDPI, Springer, ScienceDirect, Google Scholar, and PubMed. This review summarizes and documents the results by organizing the nutritional composition, chemical composition, and pharmacological activity in a table using some of the collected literature.

## 3. Composition of *S. aethiopicum*

### 3.1. Nutrient Compositions

Eggplant is the most widely consumed vegetable in the world, and three major types of eggplant are cultivated worldwide: *Solanum macrocarpon* L. (*S. macrocarpon*), *Solanum melongena* L. (*S. melongena*), and *S. aethiopicum*. Of these, *S. macrocarpon* cannot be eaten because it contains unsafe levels of glycoalkaloids (α-solamargine and α-solasonine), which is why it is primarily used as an ornamental plant. In contrast, *S. melongena* and *S. aethiopicum* are edible as they contain either significantly lower or negligible levels of these toxic substances [15].

Chinedu et al. evaluated the nutritional composition of *S. aethiopicum* purchased from the Mile 12 market in Lagos, Southwestern Nigeria [13]. Ikpeazu et al. confirmed the nutritional information of *S.aethiopicum* leaves [16]. The analysis of the fruits and leaves was conducted using the official methods established by the Association of Official Analytical Chemists. The fruit of *S. aethiopicum* contained 89.27 ± 0.12% moisture and 4.14 ± 0.11%, 2.24 ± 0.03%, and 0.52 ± 0.04% of the three major nutrients of carbohydrates, proteins, and fats, respectively. The leaves of *S. aethiopicum* also contained 84.43 ± 3.09% moisture and 4.26 ± 0.80%, 6.92 ± 1.04%, and 0.79 ± 0.16% carbohydrates, proteins, and fats, respectively [13,16]. These data are summarized in Table 1. *S. aethiopicum* varies slightly depending on the cultivar and the growing region, but the nutritional composition remains relatively constant [5,17].

*S. aethiopicum* is rich in dietary fiber, which helps maintain a healthy digestive system by relieving constipation and abdominal bloating. Additionally, its low soluble carbohydrate content reduces the body’s absorption of glucose, thereby lowering blood sugar levels. The high fiber and low calorie contents of *S. aethiopicum* are believed to significantly contribute to body fat reduction. Given these characteristics, *S. aethiopicum* may induce rapid satiety and potentially decrease the frequency of food cravings [18].

### 3.2. Microelements Compositions

In a study conducted by Han et al. [5], the concentrations of calcium, magnesium, iron, potassium, sodium, zinc, and phosphorus in dried fruits were measured, yielding values of 0.310, 0.595, 0.025, 4.475, 0.865, 0.077, and 1.091 mg/g, respectively. Additionally, Ikpeazu et al. [16] reported the concentrations of these minerals through spectrophotometric tests in dried leaves as 25.81 ± 1.86, 2.92 ± 0.62, 13.50 ± 1.40, 67.60 ± 4.91, 5.29 ± 0.94, 0.82 ± 0.05, and 7.23 ± 0.60 mg/100 g, respectively.

In a recent study, Yaméogo et al. [19] analyzed the concentrations of calcium, magnesium, iron, potassium, sodium, zinc, and phosphorus in the dried leaves and fruits of *S. aethiopicum* following the sample’s mineralization through humid digestion, as described by Houba et al. [20]. The results obtained were as follows: for fruits: 126 ± 7, 187 ± 14, 21 ± 1, 3582 ± 20, 87 ± 10, 2 ± 1, and 29 ± 5 (means ± SDs, mg/100 g); for leaves: 1048 ± 56, 666 ± 10, 12 ± 2, 3064 ± 81, 77 ± 16, 20 ± 3, and 327 ± 91 (means ± SDs, mg/100 g), respectively. The study of these components is summarized in Table 2.

Anemia is a blood disease that occurs when the number of healthy red blood cells (RBCs) in circulation decreases, preventing the body’s tissues from receiving sufficient oxygen. It can be caused by deficiencies in iron or vitamins. The iron abundant in the leaves of *S. aethiopicum* is known to help prevent anemia by stimulating the production of red blood cells.

Moreover, *S. aethiopicum* is a nutraceutical that has many therapeutic and health effects, including improving glaucoma patients’ visual function. Calcium intake through *S. aethiopicum* is recognized for its role in strengthening bones, increasing bone mineral density, regulating body fluids, and performing various other functions essential for optimal bone and muscle health. Numerous studies have demonstrated that the consumption of *S. aethiopicum* can help mitigate bone loss, osteoporosis, and other bone-related disorders [18].

### 3.3. Phytochemical Compositions

*S. aethiopicum* contains various phytochemicals such as alkaloids, flavonoids, saponins, cardiac glycosides, anthocyanins, tannins, anthraquinones, and phenols [9,13,14,17,21,22,23,24,25,26,27,28,29,30]. Alkaloids exhibit a mild suppressive effect on the central nervous system, leading to analgesic properties. Saponins possess antimicrobial activity that provides protection against various microorganisms and pathogens. Anthocyanins and flavonoids are recognized as potent antioxidants; in particular, flavonoids have demonstrated lipid-lowering, antibacterial, anti-inflammatory, and anticancer effects, in addition to their antioxidant capabilities [13]. Numerous studies have conducted phytochemical analyses using high-performance liquid chromatography or gas chromatography, while also exploring the biological effects of *S. aethiopicum*. The results of these phytochemical analyses are presented in Table 3 [9,21,23,26,30].

## 4. Pharmacological Effects

### 4.1. Anti-Inflammatory Effect

Inflammation is the body’s defense response that removes damaged tissue and creates new tissue [31]. It is also a defense mechanism against infectious agents, antigenic attacks, and physical, chemical, or traumatic damage. However, inflammation can also cause, prolong, or worsen many diseases [32].

The anti-inflammatory effects of *S. aethiopicum* have been reported in several studies [25,33,34,35]. For example, Anosike et al. [33] administered methanol extract of *S. aethiopicum* fruit to Wistar rats both orally and intravenously. They assessed its anti-inflammatory properties by inducing paw edema with egg albumin and using the cotton pellet method to induce granuloma. Next, they injected *S. aethiopicum* methanol extract (100, 200, and 400 mg/kg) intraperitoneally and compared it with a control group. Their results showed that the edema was significantly (*p* < 0.05) reduced in the treatment group. Paw edema was also significantly (*p* < 0.05) reduced when *S. aethiopicum* was included in the diet of Wistar rats at concentrations of 5%, 10%, and 20% for 14 days. Cotton pellet-induced granuloma was evaluated by aseptically transplanting high-pressure sterilized cotton pellets on both sides, administering various concentrations of *S. aethiopicum* extract (100, 200, and 400 mg/kg), and orally administering indomethacin (10 mg/kg) to rats for seven days. The administration of *S. aethiopicum* methanol extract significantly (*p* < 0.001) reduced granuloma tissue formation comparable to the effect of indomethacin (10 mg/kg), a non-steroidal anti-inflammatory (NSAID) drug.

Anosike et al. [25] also evaluated leukocyte migration and vascular permeability in rats and human erythrocyte membrane stabilization in experimentally induced inflammation and found that the methanol extract of *S. aethiopicum* reduced (*p* < 0.05) acetic acid-induced vascular permeability and agar-induced leukocyte mobilization.

Inflammation initially causes vasodilation and increased vascular permeability, along with cell activation. Anosike et al. induced vasodilation and increased vascular permeability by intraperitoneally injecting 0.6% acetic acid in rats. Administering the *S. aethiopicum* methanol extract reduced vascular permeability. In addition, the absorbance value of the peritoneal fluid was measured to assess the leakage of body fluid into the abdominal cavity across the vascular epithelial wall. The reduction of Evans blue by the *S. aethiopicum* extract indicated a decrease in body fluid leakage. Additionally, hemolysis of human red blood cells (HRBCs) induced by heat and hypotonicity was inhibited in a dose-dependent manner when treated with *S. aethiopicum* methanol extract (400, 600, and 800 μg/mL). Similarly, Adedoyin evaluated the anti-inflammatory effect of the ethanol extract of *S. aethiopicum* leaves on rat red blood cells [34], noting that the red blood cell membrane is similar to the lysosomal membrane, which is associated with the inflammatory response. Consequently, anti-lipoxygenase activity was observed at concentrations of 20, 40, 60, 80, and 100 μg/mL, with the highest activity noted at 100 μg/mL (*p* < 0.05).

Representative cytokines related to inflammation include tumor necrosis factor alpha (TNF-α) and interleukin-6 (IL-6) [36]. According to a study by Lela et al. [35], mRNA expression of TNF-α and IL-6 was reduced (*p* < 0.001) in RAW264.7 cells treated with *S. aethiopicum* peel extract. This reduction was related to kaempferol-3-O-rutinoside, a flavanol found in *S. aethiopicum* peel extract, which has been shown to improve inflammatory processes by targeting the toll-like receptor 4/nuclear factor-kappa B (TLR4/NF-κB) pathway.

### 4.2. Antidiabetic and Anti-Obesity Effects

Obesity is a multifactorial disease, which is currently one of the top ten health problems in the world, affecting more than one-third of the world’s population. It is caused by an imbalance between energy consumption and intake, adversely affecting health by leading to various complications such as type 2 diabetes and cardiovascular disease [37]. Various methods for managing obesity exist, including exercise, diet therapy, surgery, and drug treatment. However, drug treatments for obesity are hampered by limited effectiveness, high costs, and potential side effects [38]. Therefore, alternative treatments using natural products or drugs based on natural products that are safe and effective for obesity treatment have been under consideration for several decades [39,40,41,42].

Akinwunmi and Ajibola [27] administered a “cafeteria diet” (high-carbohydrate diet) of commercial food pellets as a control group to 42-week-old female Wistar rats. The treatment group was also treated with an extract of the fruit of *S. aethiopicum* with 80% (*v*/*v*) acetone and ethyl acetate, while 50 mg/kg of orlistat was used as a positive control group for comparison. The treatment with *S. aethiopicum* resulted in decreased (*p* < 0.01) body weight, blood sugar, total cholesterol (TC), triglycerides (TG), low-density lipoprotein cholesterol (LDL-C), very-low-density lipoprotein cholesterol (VLDL-C), the liver enzymes aspartate transaminase (AST), alanine transaminase (ALT), atherosclerosis index atherogenic coefficient (AC), and cardiac risk ratio and increased (*p* < 0.01) high-density lipoprotein cholesterol (HDL-C), creatinine, total protein, and albumin. These findings suggested that the phenolic component *S. aethiopicum* was able to effectively prevent obesity.

Obesity and diabetes are closely linked, as evidenced by the specific hypoglycemic effects observed in anti-obesity studies [43]. The goals of diabetes management are to normalize blood glucose levels, maintain sustainable medication requirements, and, ultimately, improve outcomes and prolong life. Socioeconomically disadvantaged diabetic patients can face financial difficulties because of the medication costs involved in diabetes management, meaning that providing cost-effective and easily accessible dietary options and resources is often necessary. In this regard, utilizing natural products for glycemic control would be beneficial [6], and several studies have already validated the antidiabetic effects of *S. aethiopicum*.

For example, Anyakudo et al. [6] examined blood glucose levels and body weight in adult male Wistar rats induced with diabetes for up to six weeks. They conducted an oral glucose tolerance (OGT) test and performed gross and histological analyses of the pancreas using hematoxylin and eosin (H&E) staining techniques. The study revealed that rats given the fruit and extract of *S. aethiopicum* experienced a decrease in average body weight gain and blood glucose concentration. Furthermore, their lipid profiles and blood glucose tolerance improved and the structure of their pancreatic tissue remained stable.

Nwanna et al. [21] evaluated the antidiabetic effect of *S. aethiopicum* by using diabetic male Wistar rats induced with streptozotocin at a concentration of 35 mg/kg. The rats were fed on a diet containing 20–40% of *S. aethiopicum* for 14 days, which led to a reduction (*p* < 0.05) in blood sugar levels and carbohydrate hydrolyzing enzymes, such as pancreatic α-amylase and intestinal α-glucosidase. Additionally, angiotensin-1-converting enzyme activity, a major contributor to cardiovascular diseases like diabetes and hypertension, was shown to have decreased (*p* < 0.05). These results demonstrated similar decreases to rats in the positive control group, which were treated with 100 mg/kg metformin.

One study evaluated the hypoglycemic effects of fruit ethanol extract from *S. aethiopicum* using rabbits [44]. The evaluation was conducted on control rabbits as well as those administered with 80 mg/kg of alloxan. This group was compared to another group that received 400 mg/kg of the extract and 200 mg/kg of tolbutamide, a standard drug known for its hypoglycemic efficacy. In rabbits with normal blood sugar levels, administering *S. aethiopicum* extract resulted in a 49.1% reduction (*p* < 0.05) in blood sugar levels after three hours. In the rabbits treated with alloxan, the extract exhibited a hypoglycemic effect of 36.9%, compared with a 54.2% hypoglycemic effect observed following the administration of tolbutamide.

The antidiabetic effects of *S. aethiopicum* have been confirmed in the leaves and peel of the fruit as well as the fruit itself. According to a study by Tuem et al. [45], the aqueous and methanol extracts of *S. aethiopicum* leaves were shown to help reduce body weight and manage diabetes. When the extract was administered to alloxan-induced diabetes rats at 200 or 300 mg/kg, the fasting blood glucose measurement results were reduced by −61.38% and −60.60% in aqueous and methanol extracts of the 300 mg/kg group, respectively, which was higher than that of the antidiabetic drug glibenclamide by −55.65%. These effects are explained by various phytochemicals present in *S. aethiopicum* such as flavonoids, terpenoids, saponins, polyphenols, alkaloids, tannins, and cardiac glycosides.

Okafor et al. [46] conducted a study to evaluate the antidiabetic effects of *S. aethiopicum* using male Wistar rats with streptozotocin-induced diabetes. The researchers administered a methanol extract of *S. aethiopicum* leaves at concentrations of 200, 400, and 600 mg/kg and compared it with the effects of 2 mg/kg of glibenclamide, a drug that stimulates pancreatic beta cells to enhance insulin secretion, which served as a positive control. The results showed that *S. aethiopicum* extract caused a reduction (*p* < 0.05) in blood glucose and plasma lipid levels when administered orally, as well as an increase in insulin secretion by restoring pancreatic beta cells. The researchers attributed these effects to the presence of phenolics, condensed tannins, gallotannins, and flavonoids in *S. aethiopicum*.

Recently, Ponticelli et al. [9] evaluated the antidiabetic and anti-obesity effects of ethanol extracts from the peel and whole fruit of *S. aethiopicum* from the Basilicata region of Italy. They confirmed that the activities of α-glucosidase, which are related to carbohydrate degradation and absorption, were strongly inhibited (*p* < 0.05) by *S. aethiopicum* peel extract (50% inhibitory concentration (IC_50_) values of peel and whole fruit were 0.0535 ± 0.0118 mg/mL and 1.532 ± 0.292 mg/mL, respectively). Notably, the inhibition ability of the extract was greater than that of acarbose, an inhibitor of α-glucosidase. Additionally, the extract inhibited (*p* < 0.05) the activity of aldose reductase (AR), with the peel extract exhibiting an IC_50_ of 0.974 ± 0.128 mg/mL and the whole fruit extract exhibiting an IC_50_ of 0.789 ± 0.05 mg/mL. AR is known to contribute to diabetic complications. *S. aethiopicum* peel extract demonstrated that it could significantly increase (*p* < 0.05) weight loss and improve glucose and lipid metabolism in a C57BL/6J male mouse with high-fat-diet-induced obesity and diabetes. The extract improved (*p* < 0.05) lipid levels (reduced TC, decreased LDL-C, and increased HDL-C) and upregulated (*p* < 0.05) the expression of AMP-activated protein kinase (AMPK) and glucose transporter type 4 (GLUT4) in the liver and adipose tissue. To summarize, several studies have demonstrated that *S. aethiopicum* could provide a promising natural alternative to treating obesity and diabetes.

### 4.3. Anxiolytic Effect

Stressful situations can lead to an increase in prostaglandin E2 synthesis in the hypothalamus, resulting in elevated body temperature. Reducing the production of prostaglandins, therefore, can elucidate an antipyretic effect. Several studies have demonstrated that various natural products counteract stress-induced hyperthermia by exerting anxiolytic effects [47,48].

Tekwem et al. [49] used specific evaluation criteria to confirm the anxiolytic and antipyretic effects of a water-soluble extract of *S. aethiopicum* leaves in Swiss mice. First, they used a stress-induced body temperature test to assess the impact of *S. aethiopicum* on body temperature increases in stressful situations. The results showed that mice given a dose of 175 mg/kg of *S. aethiopicum* exhibited a significant reduction (*p* < 0.001) in stress-induced hyperthermia. Second, they conducted an elevated plus-maze test and found that the mice that received 175 mg/kg of *S. aethiopicum* exhibited a significant increase (*p* < 0.01) in both the time spent in the open arm and the number of entries conducted. This was significant as anxious mice typically spend less time in the open arm because of fear of exposure. Third, when they assessed the activity level of the mice using the open arena test, the mice that had been given *S. aethiopicum* showed an increase in activities (*p* < 0.01) such as grooming and crossing, which is known to reduce anxiety.

During the hole-plate test, the mice exhibited a high number of head tilts and an increase in (*p* < 0.001) exploratory behavior, which indicates that *S. aethiopicum* has an anxiolytic effect and possesses sedative properties.

Similarly, Abubakar et al. [50] conducted a study where a methanol extract of *S. aethiopicum* fruit was administered at doses of 25, 50, and 100 mg/kg along with diazepam as a positive control (0.5 mg/kg) to assess the anxiolytic effect of *S. aethiopicum* in Swiss mice. The study revealed similar results in the open field test and the elevated plus maze. Furthermore, in the beam walking assay, they noted that the sedative effect of the *S. aethiopicum* extract led to increased (*p* < 0.05) foot slipping and a longer time to reach the goal box. Additionally, in the ketamine-induced sleep test, the extract reduced (*p* < 0.001) the sleep onset time and increased (*p* < 0.001) total sleep duration. Collectively, these findings suggest that extracts from the fruit and leaves of *S. aethiopicum* have anxiolytic and sedative properties.

### 4.4. Antibacterial Effect

Developing herbal medicines that can replace resistant antibiotics is vital because they can be produced very cheaply. Oluremi et al. [51] evaluated the antibacterial effect of the fruits, leaves, and methanol extract of *S. aethiopicum* along with commercial drugs by disk diffusion and agar well diffusion methods. They also performed minimum inhibitory concentration (MIC) determination to assess different concentrations of the extract and determined the lowest concentration at which microbial growth was visually inhibited. *S. aethiopicum* methanol extract exhibited antibacterial effects against bacteria, fungi, and pathogens, exhibiting significant, concentration-dependent antibacterial effects against *Staphylococcus aureus*, *Pseudomonas aeruginosa*, *Escherichia coli*, *Klebsiella* sp., and *Salmonella typhi*. The respective inhibition diameters in 200 mg/mL were confirmed as 15.9 ± 0.13, 19.8 ± 0.17, 15.0 ± 0.23, 15.1 ± 0.22, and 16.7 ± 0.17 mm. In addition, MIC was shown at 1.56 mg/mL or 3.13 mg/mL for all strains. Oluremi et al. stated that the antibacterial effect of *S. aethiopicum* was attributed to the abundance of alkaloids and polyphenols, which demonstrated strong in vitro antibacterial activity against pathogenic microorganisms.

Eze et al. [52] conducted a study to determine the protective effect of an ethanol extract of *S. aethiopicum* leaves on *E. coli*-induced pyelonephritis. The test organisms used in the study were isolated from urine samples of infected patients. The *S. aethiopicum* ethanol extract exhibited enhanced antibacterial activity with increasing concentrations (50, 100, 200, and 400 mg/mL). The leaf extract of *S. aethiopicum* demonstrated mean inhibition zone diameters of 14.00 mm, 12.50 mm, 11.80 mm, and 10.60 mm against *E. coli*, *Staphylococcus aureus*, *Streptococcus* sp., and *Klebsiella* sp., respectively. Their study demonstrated that the antimicrobial phytochemicals present in *S. aethiopicum* could assist in influencing and restoring the cellular function and structural integrity of the kidney. These results suggest that *S. aethiopicum* could be a potential source of safe and inexpensive agents for treating bacterial infections.

### 4.5. Anticancer Effect

Cancer is a global health problem characterized by uncontrolled, abnormally rapid cell growth [53]. Cancer cells invade and destroy surrounding healthy tissue in the body [29]. Attempts to cure cancer have been pursued through various means, including the use of plant-derived anticancer agents currently being tested in clinical trials. Representative examples include vinblastine, vincristine, topotecan, irinotecan, and etoposide, which were all developed from plant and other natural compounds [54]. Plant-derived compounds play an important role in the discovery and development of various clinically useful drugs, including anticancer agents [55]. Flavonoids, terpenoids, and anthraquinones present in *S. aethiopicum* have been shown to regulate reactive oxygen species (ROS) scavenging enzyme activity, arrest the cell cycle, and induce apoptosis [51], all of which can inhibit the proliferation and invasiveness of cancer cells.

The anticancer activity of *S. aethiopicum* was confirmed in several studies using Jurkat cells [56,57]. Kouassi et al. [57] evaluated the anticancer activity of the n-butanol fraction of the flower stalk of *S. aethiopicum* obtained from the methanol extract, which demonstrated an IC_50_ value of 11.77 ± 2.4 mg/mL, inducing antiproliferative and apoptotic activities. Oluremi et al. [51] also assessed the anticancer activity of *S. aethiopicum* using the MTT assay in cervical cancer (HeLa), breast cancer (MCF-7), and epidermoid carcinoma of the larynx (HEp-2). The *S. aethiopicum* extract exhibited cytotoxicity (CC_50_) of 79.97, 83.03, 87.20, and 78.01 μg/mL in HeLa cells with four extract solvents: hexane, dichloromethane, ethyl acetate, and methanol. In HEp-2 cells, it had a CC_50_ of 47.97, 56.79, 57.02, and 53.97 μg/mL and, in MCF-7 cells, the CC_50_ was 48.87, 56.86, 38.79, and 62.57 μg/mL, respectively.

### 4.6. Antioxidant Effect

Reactive oxygen species (ROS) are reactive derivatives of oxygen produced within the human body and are often generated during cellular oxygen metabolism. Oxidative damage occurs when free radicals surpass cellular defense mechanisms. Oxidative stress, induced by free radicals, is linked to the development of many diseases such as diabetes, atherosclerosis, acute renal failure, hypertension, neurodegeneration, inflammation, autoimmune diseases, cataracts, cancer, and Parkinson’s disease [28,58]. Preventing oxidative stress by utilizing natural sources rich in antioxidants can assist in preventing many common diseases [26,59].

*S. aethiopicum*, which contains various phytochemicals, can be expected to demonstrate high antioxidant effects [60], particularly because of its abundance of phenolic compounds and flavonoids [26]. *S. aethiopicum* contains various components that can be used as natural antioxidants, such as ascorbic acid, carotenoids, and anthocyanins [61]. Consequently, many researchers have conducted various assays to evaluate the antioxidant effects of *S. aethiopicum*. Studies utilized various methods such as the DPPH (2,2-diphenyl-1-picrylhydrazyl) radical scavenging assay [26,28,58,59,62,63,64,65,66,67], the ABTS (2,2′-azino-bis(3-ethylbenzothiazoline)-6-sulfonic acid) radical scavenging assay [28,66], intracellular ROS determination [26,35], the hydroxyl radical scavenging activity assay [28,59,67], the hydrogen peroxide scavenging activity assay [67], Fe^2+^ induced lipid peroxidation [28], the FCA (Fe^2+^ chelating activity) [67], the FRAP (ferric reducing antioxidant power) assay [26,63,64,66,67], the ORAC (oxygen radical absorbance capacity) assay [26], and the PMA (phosphomolybdenum assay) [67] to assess the antioxidant properties of the leaves, stalks, and fruits of *S. aethiopicum*. Their findings indicated that *S. aethiopicum* exhibited significant antioxidant activity.

### 4.7. Other Effects

#### 4.7.1. Anemia Ameliorative Effect

Ekweogu et al. [68] evaluated the effectiveness of *S. aethiopicum* in improving anemia and mitigating organ toxicity using phenylhydrazine in mice. The free radical activity of phenylhydrazine can oxidize hemoglobin and damage red blood cells, which can lead to anemia [69]. Phenylhydrazine can also potentially damage tissues and organs, including the liver and kidneys, as well as increase toxicity due to antioxidant depletion resulting from undesirable reactions with free radicals [70]. In this study, hematopoietic factors, including packed cell volume (PCV), red blood cell (RBC) indices, hemoglobin (Hb), mean corpuscular volume (MCV), and mean corpuscular hemoglobin (MCH) values, were evaluated (*p* < 0.05) in male and female rats treated with an aqueous leaf extract of *S. aethiopicum*. The results showed that the values of the various hematopoietic factors, which had decreased because of the phenylhydrazine treatment, were significantly elevated following the administration of the extract. These improved parameters may be attributable to enhanced levels of hematopoiesis and erythroid production in the bone marrow, facilitated by the high levels of essential minerals (Fe^2+^, Zn^2+^, and Cu^2+^) and vitamins (A, C, E, and B), as well as the substantial presence of antioxidant compounds such as polyphenols and flavonoids present in *S. aethiopicum*.

#### 4.7.2. Anti-Hyperlipidemic Effect

Ekweogu et al. [68] evaluated the anti-hyperlipidemic effects of an aqueous leaf extract of *S. aethiopicum*. The plasma levels for TC, TG, LDL-C, and VLDL-C in rats after being administered aqueous leaf extract of *S. aethiopicum* were significantly reduced (*p* < 0.05) compared with the control group. Additionally, HDL-C levels increased significantly compared with the negative control group (*p* < 0.05). In addition, Atukpa et al. [71] observed improvements in blood lipid profiles including TG, LDL-C, HDL-C, and TC, as well as a reversal of hyperlipidemia following the administration of *S. aethiopicum* fruit extract. They suggested that the extract’s potent antioxidant properties contributed to these improvements by inhibiting free radical production. Both these studies indicate that *S. aethiopicum* possesses anti-hyperlipidemic properties.

#### 4.7.3. Renoprotective Effect

It is well known that serum levels of urea, creatinine, sodium, chloride, bicarbonate, potassium, and inorganic phosphate are elevated in cases of renal dysfunction [72]. A study conducted by Ekweogu et al. [68] demonstrated that treatment with *S. aethiopicum* extract significantly reduced (*p* < 0.05) levels of urea, creatinine, sodium, and potassium in both male and female rats, suggesting that *S. aethiopicum* can assist in protecting the kidneys from toxic substances. The extract appears to enhance the kidneys’ ability to excrete toxic waste products (i.e., urea and creatinine) while providing renal protection without inducing kidney damage or dysfunction.

#### 4.7.4. Hepatoprotective Effect

The liver is a vital organ responsible for xenobiotic metabolism and detoxification, bile secretion, bilirubin metabolism, and the synthesis and storage of biomolecules. Alkaline phosphatase (ALP), AST, ALT, and bilirubin levels have been shown to increase in response to liver cell damage and tissue necrosis. Ekweogu et al. [68] observed that these biomarkers decreased significantly (*p* < 0.05) following treatment with an aqueous leaf extract of *S. aethiopicum* in both male and female rats. In addition, hypertoxic changes associated with liver toxicity were noted in rats treated with phenylhydrazine. However, when administered concurrently with *S. aethiopicum*, the presence of Kupffer cells—which are crucial for the resolution of hepatic inflammation and fibrotic damage—was confirmed. Based on these findings, it can be inferred that *S. aethiopicum* possesses hepatoprotective properties.

#### 4.7.5. Fertility-Improving Effect

Male fertility is primarily determined by sperm morphology, concentration, and motility. However, studies have shown that the excessive consumption of artificial sweeteners can adversely affect male fertility [73]. The membranes of sperm cells are rich in polyunsaturated fatty acids, which are susceptible to damage from ROS since oxidative stress can disrupt the lipid matrix structure, adversely affecting sperm production and motility [74]. Artificial sweeteners, such as saccharin, can then traverse the blood–testis barrier and interfere with sperm production in germ cells [75]. Atukpa et al. [71] conducted a study on the effects of an ethanol fruit extract of *S. aethiopicum* on sperm abnormalities induced by 10 mg/kg of saccharin in male Wistar rats. The group that was given saccharin exhibited sperm cell damage, whereas the group treated with *S. aethiopicum* extract demonstrated a dose-dependent improvement in the semen parameters results (morphology, viability, concentration, motility) caused by the saccharin.

Queen et al. aimed to investigate the effects of *S. aethiopicum* leaf consumption on male fertility [76]. They evaluated the effects on testicular histology, as well as changes in gonadotropin-releasing hormones, including the follicle-stimulating hormone (FSH), luteinizing hormone (LH), and testosterone by administering doses of 100, 200, and 300 mg/kg of *S. aethiopicum* to male Wistar rats over a period of 30 days. The serum levels of FSH, LH, and testosterone all showed a significant increase (*p* < 0.05) in a dose-dependent manner. Additionally, when extract administration was discontinued, the serum levels of reproductive hormones such as FSH, LH, and testosterone either decreased or were reversed. This phenomenon can be attributed to the high content of saponins, which enhance testosterone levels, and flavonoids, which improve androgen levels.

The researchers also compared the testicular histology of mice that were administered the extract for 30 days with that of a control group and found that the extract not only improved sperm production but also increased the number of sperm-producing cells. They determined that this effect was due to the cytoprotective properties of flavonoids, which function as free radical scavengers. This suggests that *S. aethiopicum* can enhance fertility in addition to being a potent antioxidant.

## 5. Conclusions

This review has demonstrated that *S. aethiopicum* possesses numerous pharmacological and biological properties, including anti-inflammatory, antidiabetic, anti-obesity, anxiolytic, antibacterial, anticancer, and antioxidant effects, highlighting its potential as an alternative therapy in treating a variety of illnesses. The pharmacological and biological activities of *S. aethiopicum* are described in Table 4. Previous studies have revealed that these medicinal effects are attributed to the alkaloids, cardiac glycosides, flavonoids, phytosterols, saponins, steroids, tannins, terpenoids, and minerals, as well as other inorganic substances, that are abundantly present in *S. aethiopicum*. Current studies primarily focus on in vitro and in vivo experiments using extracts from the fruits or leaves of *S. aethiopicum*. However, to more accurately evaluate its efficacy and safety, additional studies, including single-compound investigations and clinical trials based on current research, are essential. It is hoped that the current studies and literature reviews will serve as a cornerstone for further research into the efficacy and safety of *S. aethiopicum* as a potential medicinal therapy.

## Figures and Tables

**Table 1:** Proximate nutritional information of *S. aethiopicum*.

Contents	Fruits [13]	Leaves [16]
Moisture	89.27 ± 0.12	84.43 ± 3.09
Crude protein	2.24 ± 0.03	6.92 ± 1.04
Fat	0.52 ± 0.04	0.79 ± 0.16
Crude fiber	2.96 ± 0.08	1.82 ± 0.37
Ash	0.87 ± 0.03	1.79 ± 0.16
Carbohydrate	4.14 ± 0.11	4.26 ± 0.80

Results are means ± SDs of triplicate determinations (%, *n* = 3).

**Table 2:** Mineral composition of *S. aethiopicum* [19].

Contents	Fruits	Leaves
Calcium (Ca)	126 ± 7	1048 ± 56
Magnesium (Mg)	187 ± 14	666 ± 10
Iron (Fe)	21 ± 1	12 ± 2
Potassium (K)	3582 ± 20	3064 ± 81
Sodium (Na)	87 ± 10	77 ± 16
Zinc (Zn)	2 ± 1	20 ± 3
Phosphorus (P)	29 ± 5	327 ± 91

Results are means ± SDs (mg/100 g).

**Table 3:** Chemical compounds found in *S. aethiopicum*.

Classification	Compound Name	Extract/Part Used	Reference(s)
Aldehyde	13-Octadecenal	MeOH extract/leaves	[23]
	2-heptanal	MeOH extract/leaves	[23]
	E-15-Heptadecenal	MeOH extract/leaves	[23]
Alkaloid	Caffeine	Hydro-ethanolic extract/leaves	[30]
Alkane	Pentadecane	MeOH extract/leaves	[23]
	1-bromo triacontane	MeOH extract/leaves	[23]
	2-methylhexacosane	MeOH extract/leaves	[23]
	Cyclohexadecane	MeOH extract/leaves, Hydro-ethanolic extract/leaves	[23,30]
	Cyclotetradecane	MeOH extract/leaves	[23]
	Dotriacontane	MeOH extract/leaves	[23]
	Eicosane	MeOH extract/leaves	[23]
	Heneicosane	MeOH extract/leaves	[23]
	Heptadecane	MeOH extract/leaves	[23]
	Hexadecane	MeOH extract/leaves	[23]
	Nonadecane	MeOH extract/leaves	[23]
	Octacosane	MeOH extract/leaves	[23]
	Octadecane	MeOH extract/leaves	[23]
	Tetradecane	MeOH extract/leaves	[23]
	Tetrapentacontane, 1,54dibromodecane	MeOH extract/leaves	[23]
	Undecane	MeOH extract/leaves	[23]
	1-Docosene	MeOH extract/leaves	[23]
	1-Nonadecene	MeOH extract/leaves	[23]
Alpha- and beta-adrenergic agonist	Epinephrine	MeOH extract/leaves	[23]
Amine	7H-purin-6-amine	MeOH extract/leaves	[23]
Carboxylic acid	7-methyl-Z-tetradecen-1ol acetate	MeOH extract/leaves	[23]
	Cyanoacetic acid	MeOH extract/leaves	[23]
	Dichloroacetic acid	MeOH extract/leaves	[23]
	Trifluoroacetic acid	Hydro-ethanolic extract/leaves	[30]
Diene	1,3-Cyclopentadiene	Hydro-ethanolic extract/leaves	[30]
Ester	Carbonic acid, nonyl prop1-en-2-yl ester	MeOH extract/leaves	[23]
	Diethylsulphate	Hydro-ethanolic extract/leaves	[30]
	Pentadecyl ester (Hexanoic acid)	Hydro-ethanolic extract/leaves	[30]
Fatty acid	11-Octadecenoic acid	Hydro-ethanolic extract/leaves	[30]
	6-Octadecenoic acid	Hydro-ethanolic extract/leaves	[30]
	Cis-5-Dodecenoic acid	MeOH extract/leaves	[23]
	Decanoic acid	MeOH extract/leaves	[23]
	Oleic acid	MeOH extract/leaves	[23]
	Palmitoleic acid	MeOH extract/leaves	[23]
	Stearic acid	MeOH extract/leaves	[23]
	Hexadecanoic (palmitic) acid	Hydro-ethanolic extract/leaves	[30]
	Octadecanoic acid	Hydro-ethanolic extract/leaves	[30]
Fatty alcohol	1-heptanol	MeOH extract/leaves	[23]
	2-Hexadecen-1-ol	Hydro-ethanolic extract/leaves	[30]
	Aspidospermidine-17ol,1-acetyl-19,21-epoxy15,16-dimethoxy	MeOH extract/leaves	[23]
Heterocyclic	2-Methylpiperidine	Hydro-ethanolic extract/leaves	[30]
Phenol	4,5-di-O-Caffeoylquinic acid	MeOH extract/fruits	[21]
	4-O-Caffeoylquinic acid	MeOH extract/fruits	[21]
	5-O-Caffeoylquinic acid	MeOH extract/fruits	[21]
	Caffeic acid	MeOH extract/fruits	[26]
	Catechin	MeOH extract/fruits	[26]
	Chlorogenic acid	EtOH extract/peel, MeOH extract/fruits	[9,21]
	Delphinidin-3-rutinoside	EtOH extract/peel	[9]
	Ellagic acid	EtOH extract/peel, MeOH extract/fruits	[9,21]
	Epicatechin	EtOH extract/peel, MeOH extract/fruits	[9,21]
	Gallic acid	EtOH extract/peel, MeOH extract/fruits	[9,21]
	Isoquercitrin	MeOH extract/fruits	[26]
	Kaempferol	EtOH extract/peel, MeOH extract/fruits	[9,21]
	Kaempferol-3-O-glucoside	MeOH extract/fruits	[26]
	Kaempferol-3-O-rutinoside	MeOH extract/fruits	[26]
	L-Furan *	Hydro-ethanolic extract/leaves	[30]
	Naringenin	MeOH extract/fruits	[26]
	Naringenin-7-O-glucoside	MeOH extract/fruits	[26]
	Quercetin	EtOH extract/peel, MeOH extract/fruits	[9,21]
	Quercetin-3-O-rutinoside	MeOH extract/fruits	[26]
	Rutin	EtOH extract/peel, MeOH extract/fruits	[9,21]
Phthalates	Dibutyl phthalate	MeOH extract/leaves	[23]
Polyol	1,2,3-Propanetriol	Hydro-ethanolic extract/leaves	[30]
	ascorbic acid	EtOH extract/peel	[9]
Precursor to hormone	Estra-1,3,5 (10)-trien-17beta-ol	MeOH extract/leaves	[23]
Sesquiterpenoid	Solavetivone	EtOH extract/peel	[9]
Terpenoid	D-Limonene	Hydro-ethanolic extract/leaves	[30]
	Lycopene	EtOH extract/peel	[9]
	β-carotene	EtOH extract/peel	[9]
Triterpenoid	Squalene	MeOH extract/leaves	[23]

* 4-(5-hexylfuran-2-yl)-2-methoxyphenol.

**Table 4:** Summary of pharmacological effects of *S. aethiopicum*.

Pharmacological Effect(s)	Test Substance (Dose)	Study Model	Results	Ref.
Anti-inflammatory effect	Fruit methanol extract (100, 200, and 400 mg/kg)	Swiss albino mice, Wistar rats	Paw edema ↓; Granuloma tissue formation ↓	[33]
	Fruit methanol extract (100, 200, and 400 mg/kg)	Swiss albino mice, Adult Wistar rats	Acetic acid induced vascular permeability and agar induced leukocyte mobilization ↓	[25]
	Leaf ethanol extract (20, 40, 60, 80, and 100 μg/mL)	Rat red blood cells	Lipoxygenase activity ↓	[34]
	Peel extract (100 and 200 μg/mL)	Caco-2 cells	TNF-α ↓; IL-6 ↓	[35]
Antidiabetic and anti-obesity effects	Fruit acetone and ethyl acetate extracts (200 and 400 mg/kg)	Female Wistar rats	TC, TG, LDL-C, VLDL-C, AST, and ALT ↓; HDL-C, Creatinine, Total protein, and Albumin ↑	[27]
	Fruit aqueous extract, supplemented diet	Male Wistar rats	Body weight gain ↓; Blood glucose ↓; Improved lipid profile and glycemic tolerance	[6]
	Fruit-supplemented diet (with 20 and 40% *S. aethiopicum*)	Male Wistar rats	α-amylase activity and α-glucosidase activity ↓; ACE activity ↓	[21]
	Fruit ethanol extract (400 mg/kg)	Rabbits	Blood sugar levels ↓	[44]
	Leaf aqueous and methanol extract (200 and 300 mg/kg)	Male Wistar rats	Fasting blood glucose ↓	[45]
	Leaf methanol extract (200, 400, and 600 mg/kg)	Male Wistar rats	Blood glucose and plasma lipid levels ↓	[46]
	Peel and fruit ethanol extract (25 mg/kg)	C57BL/6J male mice	α-glucosidase activity ↓; Aldose reductase activity ↓; Weight loss and improved glucose ↑; Improved lipid levels (TC ↓, LDL-C ↓, HDL-C ↑); AMPK and GLUT4 ↑	[9]
Anxiolytic effect	Leaf aqueous extract (17.5, 43.75, 87.5, and 175 mg/kg)	White mice, *Mus musculus* swiss	Stress-induced hyperthermia ↓;Time spent in the open arm and number of entries conducted ↑;Hole-plate test head tilts ↑	[49]
	Fruit methanol extract (25, 50, and 100 mg/kg)	Swiss mice	Amount of foot slipping and time to reach the goal box ↑; Sleep onset time ↓, Total sleep duration ↑	[50]
Antibacterial effect	Plant methanol extract (12.5, 25, 50, 100, and 200 mg/mL)	*Staphylococcus aureus*, *Pseudomonas aeruginosa*, *Escherichia coli*, *Klebsiella* sp., and *Salmonella typhi*	Diameter zone in 200 mg/mL: 15.9 ± 0.13, 19.8 ± 0.17, 15.0 ± 0.23, 15.1 ± 0.22, and 16.7 ± 0.17 mm, respectively	[51]
	Leaf ethanol extract (50, 100, 200, and 400 mg/mL)	*Escherichia coli*, *Staphylococcus aureus*, *streptococcus* sp., and *Klebsiella* sp.	Diameter zone in 400 mg/mL: 14.00, 12.50, 11.80, and 10.60 mm, respectively	[52]
Anticancer effect	Flower stalk methanol extract (10, 15, 25, and 50 mg/mL)	Jurkat cells	Antiproliferative and apoptotic activities ↑	[57]
	Plant extracts using hexane, dichloromethane, ethyl acetate, and methanol (0.01, 0.1, 1, 10, and 100 µg/mL)	HeLa, MCF-7, HEp-2 cells	HeLa cells: Cytotoxicity (CC_50_) 79.97, 83.03, 87.20, and 78.01 μg/mL; HEp-2 cells: CC_50_ 47.97, 56.79, 57.02, and 53.97 μg/mL; MCF-7 cells: CC_50_ 48.87, 56.86, 38.79, and 62.57 μg/mL	[51]
Anemia ameliorative effect	Leaf aqueous extract (200 and 400 mg/kg)	Male and female Wistar rats	PCV, RBC indices, Hb, MCV, and MCH ↑	[68]
Antihyperlipidemic effect	Leaf aqueous extract (200 and 400 mg/kg)	Male and female Wistar rats	TC, TG, LDL-C, and VLDL-C ↓; HDL ↑	[68]
	Fruit ethanol extract (50 and 100 mg/kg)	Male Wistar rats	TC, TG, and LDL-C ↓; HDL ↑	[71]
Renal protective effect	Leaf aqueous extract (200 and 400 mg/kg)	Male and female Wistar rats	Urea, creatinine, sodium, and potassium ↓	[68]
Hepatoprotective effect	Leaf aqueous extract (200 and 400 mg/kg)	Male and female Wistar rats	ALP, AST, ALT, and bilirubin levels ↓	[68]
Fertility-improving effect	Fruit ethanol extract (50 and 100 mg/kg)	Male Wistar rats	Semen parameters results ↑ (morphology, viability, concentration, motility)	[71]
	Leaf hydro-ethanolic extract (100, 200, and 300 mg/kg)	Male Wistar rats	FSH, LH, and testosterone ↑	[76]

ACE, angiotensin-1-converting enzyme; ALT, alanine transaminase; ALP, alkaline phosphatase; AMPK, adenosine monophosphate-activated protein kinase; AST, aspartate transaminase; FSH, follicle-stimulating hormone; GLUT4, glucose transporter type 4; Hb, hemoglobin; HDL-C, high-density lipoprotein cholesterol; IL-6, interleukin-6; LDL-C, low-density lipoprotein cholesterol; LH, luteinizing hormone; MCH, mean corpuscular hemoglobin; MCV, mean corpuscular volume; PCV, packed cell volume; RBC, red blood cell; TC, total cholesterol; TG, triglycerides; TNF-α, tumor necrosis factor alpha; VLDL-C, very-low-density lipoprotein cholesterol. ↑, the values or effects have increased; ↓, the values or effects have decreased.

## References

[1] Gurib-Fakim A. (2006). Medicinal Plants: Traditions of Yesterday and Drugs of Tomorrow. Mol. Aspects Med..

[2] Umaru I.J., Shuaibu S.I., Adam R.B., Habibu B., Umaru K.I., Haruna D.E., David B.C. (2020). Effect of Herbal Medicine and Its Biochemical Implication. Int. J. Adv. Biochem. Res..

[3] Prashar D., Saklani S. (2011). Pharmaceutical and Economical Aspects of Medicinal Herbs: An Overview. Res. J Pharmacogn. Phytochem..

[4] Osei M., Banful B., Osei C., Oluoch M.O. (2010). Characterization of African Eggplant for Morphological Characteristics. J. Agric. Sci. Technol..

[5] Han M., Opoku K.N., Bissah N.A.B., Su T. (2021). *Solanum aethiopicum*: The Nutrient-Rich Vegetable Crop with Great Economic, Genetic Biodiversity and Pharmaceutical Potential. Horticulturae.

[6] Anyakudo M.M.C., Omogbehin N.T., Adeyomoye O.I. (2022). Beneficial Impacts of *Solanum aethiopicum* L. in Diabetes Control. Int. J. Nutr..

[7] Aguessy S., Idossou R., Dassou A.G., Yêyinou Laura Estelle L., Yelome O.I., Gbaguidi A.A., Agre P.A., Dansi A., Agbangla C. (2021). Ethnobotanical Characterization of Scarlet Eggplant (*Solanum aethiopicum* L.) Varieties Cultivated in Benin (West Africa). J. Agric. Food Res..

[8] Chioma A., Obiora A., Chukwuemeka U. (2011). Does the African Garden Egg Offer Protection against Experimentally Induced Ulcers?. Asian Pac. J. Trop. Med..

[9] Ponticelli M., Hidalgo-García L., Diez-Echave P., Vezza T., Romero M., Robles-Vera I., Duarte J., De Biasio F., Gorgoglione D., Lela L. (2023). *Solanum aethiopicum* L. from the Basilicata Region as a Source of Specialized Metabolites with Promising Anti-Obesity Effects: Phytochemical Characterization and in Vivo Investigation in High Fat Diet-Fed Mice. Front. Pharmacol..

[10] Lester R., Daunay M.-C. (2003). Diversity of African Vegetable Solanum Species and Its Implications for a Better Understanding of Plant Domestication. Rudolf Mansfeld Plant Genet. Resour..

[11] Plazas M., Andújar I., Vilanova S., Gramazio P., Herraiz F.J., Prohens J. (2014). Conventional and Phenomics Characterization Provides Insight into the Diversity and Relationships of Hypervariable Scarlet (*Solanum aethiopicum* L.) and Gboma (*S. Macrocarpon* L.) Eggplant Complexes. Front. Plant Sci..

[12] Lester R.N. (1986). Taxonomy of scarlet eggplants, *Solanum aethiopicum* L.. Acta Hortic..

[13] Chinedu S.N., Olasumbo A.C., Eboji O.K., Emiloju O.C., Dania D.I. (2011). Proximate and Phytochemical Analyses of *Solanum aethiopicum* L. and *Solanum macrocarpon* L. Fruits. Res. J. Chem. Sci..

[14] Aniobi C.C., Okeke O., Ezeh E., Okeke H.C., Nwanya K.O. (2021). Comparative Assessment of the Phytochemical and Selected Heavy Metal Levels in *Cucumis Sativus* L. and *Solanum aethiopicum* L. Fruit Sample Grown in South Eastern and North Central Regions of Nigeria Respectively. Nat. Resour..

[15] Sánchez-Mata M.-C., Yokoyama W.E., Hong Y.-J., Prohens J. (2010). α-Solasonine and α-Solamargine Contents of Gboma (*Solanum macrocarpon* L.) and Scarlet (Solanum aethiopicum L.) Eggplants. J. Agric. Food Chem..

[16] Ikpeazu V.O., Emmanuel O., Ekweogu C.N., Akara E.U., Ugbogu E.A. (2019). A Comparative Nutritional Assessments of Leaf Extracts of Ocimum Gratissimum and *Solanum aethiopicum*. Am. J. Biomed. Res..

[17] Benjamin A., Winner K., Constance N., Ahamefula E., Ijeoma E., Chimaraoke O., Nchekwube O. (2020). Comparative Study of Chemical Composition of Three Different Eggplant Fruit Species. Asian Food Sci. J..

[18] Opara E., Udourioh G.A. (2023). Garden Egg (*Solanum aethiopicum*) as a Mystical Plant in Akabo, South Eastern Nigeria: Health and Economic Implications. J. Appl. Sci. Environ. Manag..

[19] Yaméogo C.W., Savadogo B., Garanet F., Parkouda C. (2024). Comparative Study of *Solanum aethiopicum* Leaves and Fruits Nutritional Quality. Glob. J. Nutr. Food Sci..

[20] Houba V., van Vark W., Walinga I., Vander Lee J.J. (2020). Plant Analysis Procedures.

[21] Nwanna E.E., Ibukun E.O., Oboh G. (2016). Effect of Some Tropical Eggplant Fruits (*Solanum* spp.) Supplemented Diet on Diabetic Neuropathy In Experimental Male Wistar Rats In-Vivo. Funct. Foods Health Dis..

[22] Adelakun S.A., Ukwenya V.O., Ojewale A.O., Aniah J.A., Kolawole B.P. (2023). Excessive Exposure to Sodium Fluoride Impaired Spermatogenesis, Induced Hormonal and Biochemical Imbalance and Testicular Atrophy: Ameliorating Potential of Bioactive Component of *Solanum aethiopicum* Supplementation. Phytomedicine Plus.

[23] Osuji-Kalu N.C., Ene A.C., Chukwudoruo C.S. (2024). Gas Chromatography-Mass Spectroscopy and Fourier Transform Infrared Profiling of the Bioactive Compounds Present in Methanol Leaf Extract of *Solanum aethiopicum* from Imo State, Nigeria. Afr. J. Biol. Med. Res..

[24] Nwanna E.E., Ibukun E.O., Oboh G., Ademosun A.O., Boligon A.A., Athayde M. (2014). HPLC-DAD Analysis and In-Vitro Property of Polyphenols Extracts from (*Solanum aethiopium*) Fruits on α-Amylase, α-Glucosidase and Angiotensin—1-Converting Enzyme Activities. Int. J. Biomed. Sci..

[25] Anosike C.A., Obidoa O., Ezeanyika L.U. (2012). Membrane Stabilization as a Mechanism of the Anti-Inflammatory Activity of Methanol Extract of Garden Egg (*Solanum aethiopicum*). Daru J. Fac. Pharm. Tehran Univ. Med. Sci..

[26] Faraone I., Lela L., Ponticelli M., Gorgoglione D., De Biasio F., Valentão P., Andrade P.B., Vassallo A., Caddeo C., Falabella R. (2022). New Insight on the Bioactivity of *Solanum aethiopicum* Linn. Growing in Basilicata Region (Italy): Phytochemical Characterization, Liposomal Incorporation, and Antioxidant Effects. Pharmaceutics.

[27] Akinwunmi K.F., Ajibola I.O. (2018). Evaluation of Anti-obesity Potentials of Phenolic-Rich Fraction of *Solanum aethiopicum* L. and *Solanum macrocarpon* L. on Diet-induced Obesity in Wistar Rats. Eur. J. Med. Plants.

[28] Nwanna E.E., Adebayo A.A., Ademosun A.O., Oboh G. (2019). Phenolic Distribution, Antioxidant Activity, and Enzyme Inhibitory Properties of Eggplant (*Solanum aethiopicum*) Cultivated in Two Different Locations within Nigeria. J. Food Biochem..

[29] Thing L.Y., Saleena L.A.K., Ying C.L.S., Koh R.Y., Phing P.L. (2023). Phytochemical and Cytotoxic Properties of *Solanum aethiopicum* Fruit Extracts against HeLa Human Cervical Cancer Cell Line. Asia Pac. J. Mol. Biol. Biotechnol..

[30] Vz Z., Om A., So O., Dv D. (2022). Phytochemical Screening and Gc–Ms Analysis of Bioactive Compounds Present in Hydroethanolic Leaf Extract of *Solanum aethiopicum* (L). GPH-Int. J. Biol. Med. Sci..

[31] Ghasemian M., Owlia S., Owlia M.B. (2016). Review of Anti-Inflammatory Herbal Medicines. Adv. Pharmacol. Pharm. Sci..

[32] Ferrero-Miliani L., Nielsen O.H., Andersen P.S., Girardin S.E. (2007). Chronic Inflammation: Importance of NOD2 and NALP3 in Interleukin-1β Generation. Clin. Exp. Immunol..

[33] Anosike C.A., Obidoa O., Ezeanyika L.U.S. (2012). The Anti—Inflammatory Activity of Garden Egg (*Solanum aethiopicum*) on Egg Albumin—Induced Oedema and Granuloma Tissue Formation in Rats. Asian Pac. J. Trop. Med..

[34] Olasunkanmi A.A., Afuye O.O. Evaluation of Lipoxygenase and Membrane Stabilization Inhibitory Actvity of Ethanol Solanum aethiopicum (Garden Egg) Leaf Extract. Proceedings of the 5th National Conference of the School of Pure & Applied Sciences.

[35] Lela L., Russo D., De Biasio F., Gorgoglione D., Ostuni A., Ponticelli M., Milella L. (2023). *Solanum aethiopicum* L. from the Basilicata Region Prevents Lipid Absorption, Fat Accumulation, Oxidative Stress, and Inflammation in OA-Treated HepG2 and Caco-2 Cell Lines. Plants.

[36] Rincon M. (2012). Interleukin-6: From an Inflammatory Marker to a Target for Inflammatory Diseases. Trends Immunol..

[37] Saunders K.H., Umashanker D., Igel L.I., Kumar R.B., Aronne L.J. (2018). Obesity Pharmacotherapy. Med. Clin. N. Am..

[38] Bahmani M., Eftekhari Z., Saki K., Fazeli-Moghadam E., Jelodari M., Rafieian-Kopaei M. (2016). Obesity Phytotherapy: Review of Native Herbs Used in Traditional Medicine for Obesity. J. Evid.-Based Complement. Altern. Med..

[39] Li X., Zhang Y., Wang S., Shi C., Wang S., Wang X., Lü X. (2022). A Review on the Potential Use of Natural Products in Overweight and Obesity. Phytother. Res..

[40] Lee H.S., Heo C.U., Song Y.-H., Lee K., Choi C.-I. (2023). Naringin promotes fat browning mediated by UCP1 activation via the AMPK signaling pathway in 3T3-L1 adipocytes. Arch. Pharm. Res..

[41] Choi S.M., Lee H.S., Lim S.H., Choi G., Choi C.-I. (2024). Hederagenin from Hedera Helix Promotes Fat Browning in 3T3-L1 Adipocytes. Plants.

[42] Zhao X.-Y., Wang J.-Q., Neely G.G., Shi Y.-C., Wang Q.-P. (2024). Natural Compounds as Obesity Pharmacotherapies. Phytother. Res..

[43] Ruze R., Liu T., Zou X., Song J., Chen Y., Xu R., Yin X., Xu Q. (2023). Obesity and Type 2 Diabetes Mellitus: Connections in Epidemiology, Pathogenesis, and Treatments. Front. Endocrinol..

[44] Ezugwu C.O., Okonta J.M., Nwodo N.J. (2004). Antidiabetic Properties of Ethanolic Fruit Extract of *Solanium aethiopicum* L.. J. Pharm. Allied Sci..

[45] Tuem S., Ndomou M., Manz K., Nchoutpouen N., Gouado I. (2021). Effect of Aqueous and Methanolic Extracts of *Solanum aethiopicum* Linn Gilo (Solanaceae) Leaves on Body Weight and Glycemia in Rats with Alloxan-Induced Diabetes. J. Pharmacogn. Phytochem..

[46] Okafor H., Odugbemi A., Okezie C., Achebe M. (2016). Antidiabetic and Hypolipidaemic Effects of Garden Egg (*Solanum aethiopicum*) Leaf Extract in Beta-Cells of Streptozotocin Induced Diabetic Male Wistar Rats. Annu. Res. Rev. Biol..

[47] Neteydji S., Djafsia G., Taiwe G.S., Rakotonirina S.V., Rakotonirina A., Bum E.N. (2013). Anxiolytic and Antipyretic Activities in the Decoction of Leaves of Piliostigma Reticulatum. Asian J. Pharm. Health Sci..

[48] Bum E.N., Taiwe G.S., Moto F.C.O., Ngoupaye G.T., Vougat R.R.N., Sakoue V.D., Gwa C., Ayissi E.R., Dong C., Rakotonirina A. (2011). Antiepileptic Medicinal Plants Used in Traditional Medicine to Treat Epilepsy. Clinical and Genetic Aspects of Epilepsy.

[49] Tekwem H.D., Ngah E., Guiama V.D., Touo’yem W.S.N., Sidiki N., Bum E.N. (2022). Anxiolytic and Antipyretic Effects of the Aqueous Extract of the Leaves of *Solanum aethiopicum* (*Solanaceae*) in the White Mouse Mus Musculus Swiss (Muridae). GSC Biol. Pharm. Sci..

[50] Abubakar A., Sani I.H., Malami S., Yaro A. (2021). Tropical Journal of Natural Product Research Original Research Article Anxiolytic and Sedative Activities of Methanol Extract of *Solanum aethiopicum* (Linn.) Fruits in Swiss Mice. J. Nat. Prod..

[51] Oluremi B.B., Oloche J.J., Adeniji A.J. (2021). Anticancer and Antibacterial Activities of *Solanum aethiopicum* L., *Solanum macrocarpon* L. and Garcinia Kola Heckel. Trop. J. Nat. Prod. Res..

[52] Eze H., Okoli I., Ajogwu T. (2023). Effects of Ethanolic Extracts of *Solanum aethiopicum* Leaf on Haematological Parameters of Wistar Rats Induced Kidney Infections. J. Biotechnol. Biol..

[53] Vineis P., Wild C.P. (2014). Global Cancer Patterns: Causes and Prevention. Lancet.

[54] Krupa J., Sureshkumar J., Silambarasan R., Priyadarshini K., Ayyanar M. (2019). Integration of Traditional Herbal Medicines among the Indigenous Communities in Thiruvarur District of Tamil Nadu, India. J. Ayurveda Integr. Med..

[55] Cragg G.M., Newman D.J. (2005). Plants as a Source of Anti-Cancer Agents. J. Ethnopharmacol..

[56] Christian D.K.K., Anatolievna D.S.E., Akhanovna P.M.-B.J., Yves-Alain P.B. (2016). Extract-Based Microemulsion Formulation with *Solanum aethiopicum* Peduncle: Characterization by DLS, Relative Humidity and F0 Anti-Proliferative Effect on Leukemia Cancer Cells. Pharma Innov. J..

[57] Kouadio K.C., Mamyrbekova-Bekro J.A., Boua B.B., Bekro Y.-A. (2015). HPLC Phytochemical Analysis and Antiproliferative Activity of N-Butanol Fraction of *Solanum aethiopicum* L. (*Solanaceae*) from Côte d’Ivoire. Res. J. Recent Sci..

[58] Mbondo N.N., Owino W.O., Ambuko J., Sila D.N. (2018). Effect of Drying Methods on the Retention of Bioactive Compounds in African Eggplant. Food Sci. Nutr..

[59] Adedosu O.T., Badmus J.A., Adeleke G.E., Afolabi O.K., Adekunle A.S., Olabode F.J., Fakunle P.B. (2015). Oxidative damage and dietary antioxidants: The roles of extract and fractions of *Solanium aethiopicum* leaves. Can. J. Pure Appl. Sci..

[60] Eletta O.A.A., Orimolade B.O., Oluwaniyi O.O., Dosumu O.O. (2017). Evaluation of Proximate and Antioxidant Activities of Ethiopian Eggplant (*Solanum aethiopicum* L.) and Gboma Eggplant (*Solanum macrocarpon* L.). J. Appl. Sci. Environ. Manag..

[61] Mansurat A.I., Dandago M.A., Diya’udeen H.B. (2023). Phytochemical Contents and Antioxidant Potentials of Eggplants from Kano State, Nigeria: A Review. Dutse J. Pure Appl. Sci..

[62] Michel E., Rhode N., Tsiba G., Stephane W., Simplice I.O.Y., Vital M. (2024). Nutritional and Antioxidant Evaluation and Effect of Eggplant Consumption on Anthropometric and Hematologic Parameters in Wistar Rats. Food Nutr. Sci..

[63] Diatta K., Diatta W., Dior Fall A., Mbacké Dieng S.I., Ibrahima Mbaye A., Sarr A., Adjoke Adegbindin M. (2020). Evaluation of the Antioxidant Activity of Stalk and Fruit of *Solanum aethiopicum* L. (*Solanaceae*). Asian J. Res. Biochem..

[64] Sekulya S., Nandutu A., Namutebi A., Ssozi J., Masanza M., Jagwe J.N., Kasharu A., Rees D., Kizito E.B., Acham H. (2018). Effect of Post-Harvest Handling Practices, Storage Technologies and Packaging Material on Post-Harvest Quality and Antioxidant Potential of *Solanum aethiopicum* (*Shum*) Leafy Vegetable. Am. J. Food Sci. Technol..

[65] Uroko R. (2015). Dpph (1,1-Diphenyl-2-Picrylhydrazyl) Radical Scavenging Activity of Some Ethnomedicinal Plants in Nigeria. Am.-Eurasian J. Toxicol. Sci..

[66] Ukom A., Egbujor D., Nwanagba L., Okparauka I. (2023). Effect of Pretreatments and Drying Methods on The Physicochemical and Antioxidant Properties of Whole Eggplant (*Solanum aethiopicum*) Flour. Indones. Food Nutr. Prog..

[67] Sharma L. (2018). Assessment of Bioactive Compounds, Antioxidative Activity and Quantification of Phenols through HPLC in Solanum Species. Stud. ETHNO-Med..

[68] Ekweogu C.N., Ude V.C., Nwankpa P., Emmanuel O., Ugbogu E.A. (2020). Ameliorative Effect of Aqueous Leaf Extract of *Solanum aethiopicum* on Phenylhydrazine-Induced Anaemia and Toxicity in Rats. Toxicol. Res..

[69] Pandey K., Meena A.K., Jain A., Singh R.K. (2015). Molecular Mechanism of Phenylhydrazine Induced Haematotoxicity: A Review. Am. J. Phytomedicine Clin. Ther..

[70] Kehrer J.P. (1993). Free Radicals as Mediators of Tissue Injury and Disease. Crit. Rev. Toxicol..

[71] Atukpa M., Wekhe A., Idris A., Recab R. (2023). Effects of Ethanolic Fruit Extract of *Solanum aethiopicum* (L.) on Saccharin Induced Hyperlipidemia and Sperm Abnormalities in Male Wistar Rats. J. Res. Appl. Basic Med. Sci..

[72] Kong B.-H., Tan N.-H., Fung S.-Y., Pailoor J. (2016). Sub-Acute Toxicity Study of Tiger Milk Mushroom Lignosus Tigris Chon S. Tan Cultivar E Sclerotium in Sprague Dawley Rats. Front. Pharmacol..

[73] Helal E.G.E., Al-Shamrani A., Abdelaziz M.A., El-Gamal M.S. (2019). Comparison between The Effect of Sucralose and Sodium Saccharin on Some Physiological Parameters in Male Albino Rats. Egypt. J. Hosp. Med..

[74] Alahmar A.T. (2019). Role of Oxidative Stress in Male Infertility: An Updated Review. J. Hum. Reprod. Sci..

[75] Adelakun S.A., Ukwenya V.O., Akingbade G.T., Omotoso O.D., Aniah J.A. (2020). Interventions of Aqueous Extract of Solanum Melongena Fruits (Garden Eggs) on Mercury Chloride Induced Testicular Toxicity in Adult Male Wistar Rats. Biomed. J..

[76] Queen N., Ogbu O., Zabbey V. (2023). Evaluation of the hydroethanolic leaf extracts of *Solanum aethiopicum* (garden egg) on the concentration of reproductive hormones and histology of the testis of male wistar RATS. Eur. J. Biomed. Pharm. Sci..

